# Supplementary material for: A dorsomedial prefrontal cortex-based dynamic functional connectivity model of rumination
Source: Nat Commun. 2023 Jun 15;14:3540. doi: 10.1038/s41467-023-39142-9 (PMC10272121; doi:10.1038/s41467-023-39142-9)
Supplement: Supplementary file 1 — Supplementary Information [file 41467_2023_39142_MOESM1_ESM.pdf]

Supplementary Information for

A DORSOMEDIAL PREFRONTAL CORTEX-BASED DYNAMIC FUNCTIONAL CONNECTIVITY MODEL OF RUMINATION

Jungwoo Kim[1,2,3*], Jessica R. Andrews-Hanna[4,5*], Hedwig Eisenbarth[6], Byeol Kim Lux[1,2,7], Hong Ji Kim[1,2.3], Eunjin Lee[1,2,3], Martin A. Lindquist[8], Elizabeth A. Reynolds Losin[9,10], Tor D. Wager[7†], Choong-Wan Woo[1,2,3,11†]

[1]Center for Neuroscience Imaging Research, Institute for Basic Science, Suwon, South Korea [2]Department of Biomedical Engineering, Sungkyunkwan University, Suwon, South Korea [3]Department of Intelligent Precision Healthcare Convergence, Sungkyunkwan University, Suwon, South Korea [4]Department of Psychology, University of Arizona, Tucson, AZ, USA [5]Cognitive Science, University of Arizona, Tucson, AZ, USA [6]School of Psychology, Victoria University of Wellington, Wellington, New Zealand [7]Department of Psychological and Brain Sciences, Dartmouth College, Hanover, NH, USA [8]Department of Biostatistics, Johns Hopkins University, Baltimore, MD, USA [9]Department of Psychology, University of Miami, Miami, FL, USA [10]Department of Biobehavioral Health, Penn State University, State College, PA, USA [11]Life-inspired Neural network for Prediction and Optimization Research Group, Suwon, South Korea

[*] These authors contributed equally
[†] These authors jointly supervised this work

This PDF file includes:

1. Supplementary Figures 1-6
2. Supplementary Table 1-7

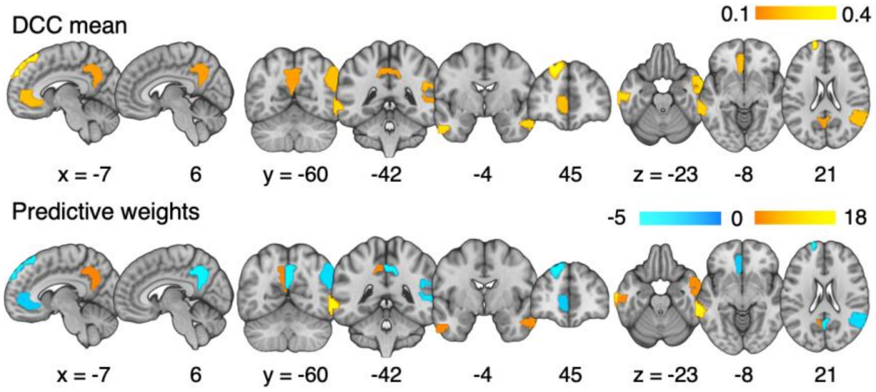

Supplementary Figure 1. DCC mean values and predictive weights in default mode network. The upper panel shows the DCC mean values of the default mode network regions with non-zero predictive weights in the original full model. The lower panel shows the same regions' predictive weights

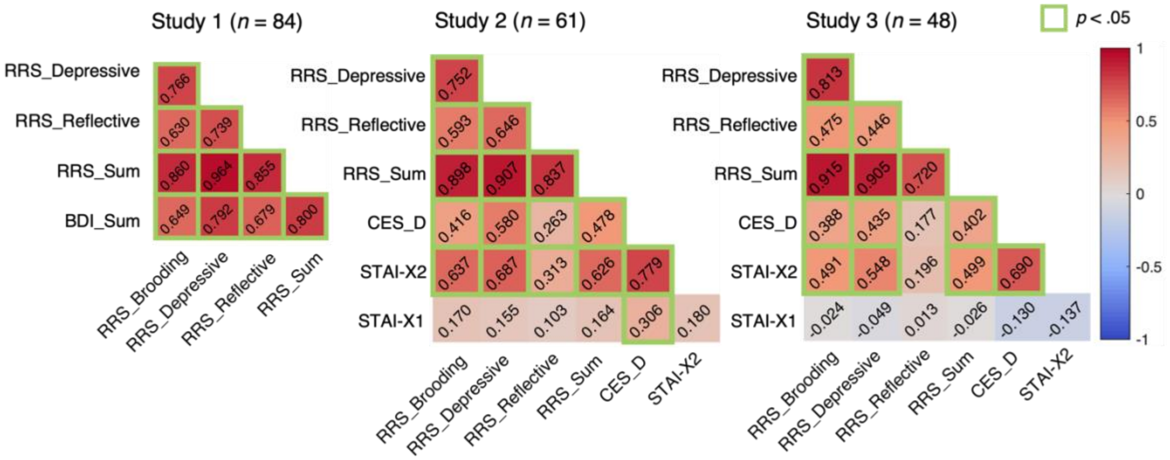

Supplementary Figure 2. Correlations among the self-report questionnaires. Heatmaps show correlations among the self-report questionnaires included in studies. Numbers in squares indicate correlation values, and the green squares mark the ones with significant correlations ($p < .05$, two-sided).

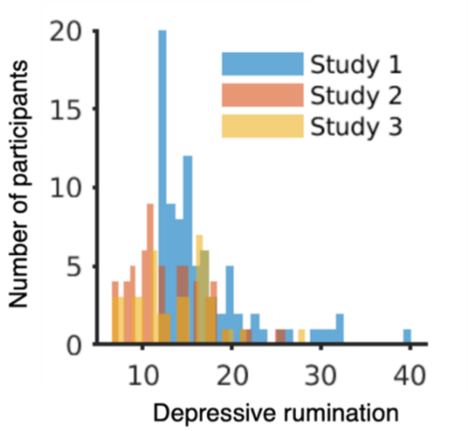

Supplementary Figure 3. Distributions of the depressive rumination scores across datasets. Study 1 dataset showed the largest variance in the depressive rumination scores. For Study 1, mean = 16.69, SD = 5.627; for Study 2, mean = 13.03, SD = 4.324; for Study 3, mean = 13.77, SD 4.323.

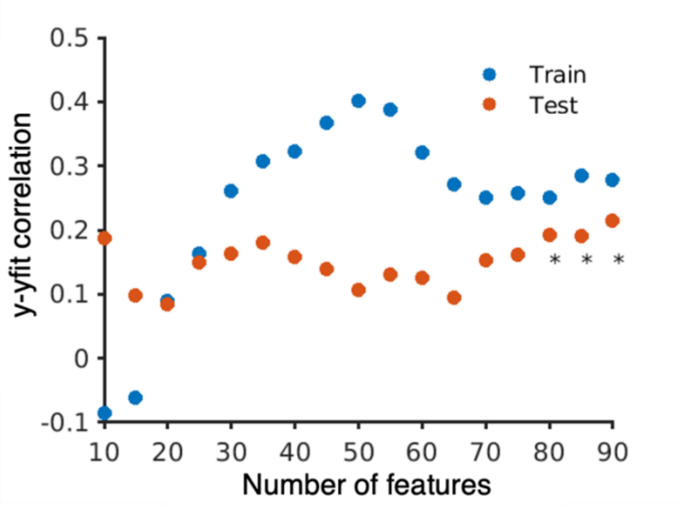

Supplementary Figure 4. Prediction performance of the dmPFC-based predictive model with varying numbers of predictors. Here we trained the dmPFC-based predictive model with a combined dataset of Studies 2 and 3 (n = 109) to examine whether the successful prediction of rumination required at least a certain number of predictors. The testing dataset was the Study 1 dataset (n = 84). We varied the number of predictors to be retained in the Lasso regression and plotted the correlation between the model response (yfit) and the dependent variable (y). For the training dataset, the y-yfit correlation was from 10-fold cross-validation. The results show that the dmPFC-based predictive model showed significant prediction performance in both training and testing datasets, but it did so only when the number of predictors was greater than 80. In the training dataset (blue dots), the p-value was 0.005 for 80 features, 0.002 for 85 features, and 0.003 for 90 features. In the testing (orange dots), the p-value was 0.038 for 80 features, 0.042 for 85 features, and 0.024 for 90 features. *$p < .05$, one-sided permutation test in both training and testing datasets.

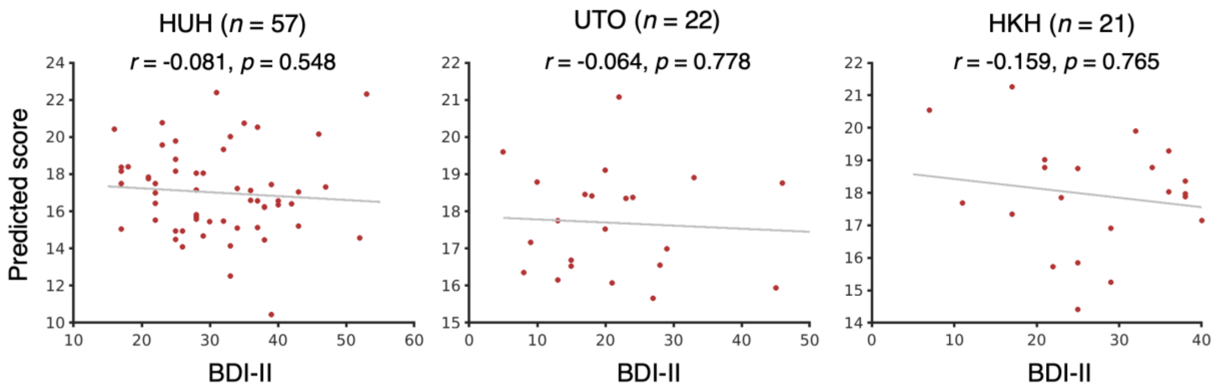

Supplementary Figure 5. Prediction results in additional clinical samples. To examine whether our model generalizes to other clinical depression datasets, we tested our model on three datasets from the Strategic Research Program for Brain Sciences (SRPBS) data that had both fMRI and behavioral (i.e., BDI-II) data. After we applied the selection criteria same as our original analysis (i.e., participants with mean framewise displacement under 0.25 and right-handed), we were able to proceed with the following datasets: a dataset from Hiroshima Kajikawa Hospital (HKH; n = 21), Hiroshima University Hospital (HUH; n = 57), and University of Tokyo (UTO; n = 22). We calculated the dmPFC-based DCC variances, applied the refined model (i.e., a model with 21 important regions), and compared the model prediction with the BDI-II scores. The results showed that our model failed to generalize in other depression datasets (HUH: r = -0.081, p = 0.548, one-sided permutation test, 95% CI [-0.348, 0.186]; UTO: r = -0.064, p = 0.778, one-sided permutation test, 95% CI [-0.514, 0.386]; HKH: r = -0.159, p = 0.765, one-sided permutation test, 95% CI [-0.622, 0.302]). There could be many reasons for this, but according to Yamashita et al.[1], the measurement bias caused by different scan parameters (esp., phase encoding direction) and MRI manufacturers could be major contributors to the generalization failure. Yamashita et al.[1] conducted detailed analyses on the SRPBS data to minimize the heterogeneity across multiple scan sites (i.e., data harmonization) and showed that phase encoding and MRI manufacturer were the two most significant contributors to the measurement bias. The COI (Center of Innovation in Hiroshima University) dataset that we included in the main manuscript was the only dataset that had the same phase encoding direction (i.e., A→P direction) and same MRI manufacturer (i.e., Siemens) as ours (i.e., Studies 1-3, Study 5). Unfortunately, no other depression datasets from the SRPBS did not use the same scan parameters and same scanner. For example, the HUH, UTO, and HKH datasets used a different phase encoding direction (i.e., P→A), and the HUH and UTO datasets used MRI scanner from a different manufacturer (i.e., GE). In our revision, we added these additional analyses and discussions on the limitation of our results.

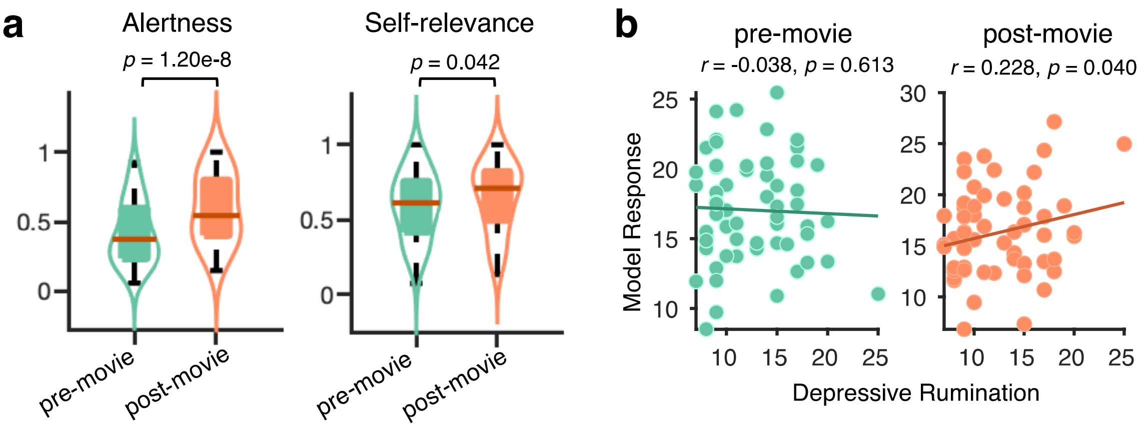

Supplementary Figure 6. Testing the model on an additional resting-state dataset. We tested our model on an additional dataset (n = 60, age = 23.35 ± 1.91 [mean ± SD], 30 males, recruited from the Suwon area, similar to Studies 2 and 3), which has an interesting experimental design feature—we administered two resting-state scans (each run was 14 minutes long) before and after participants watched a short emotional movie (9 minutes and 38 seconds long). We conducted this additional model test to further test our model's generalizability and to see whether our model showed different prediction performances depending on different resting-state conditions [2]. This movie was about a mother meeting her daughter who passed away through virtual reality, and we selected this movie to enhance internally oriented cognitive states. Specifically, we assumed that the movie would increase participants' alertness and stimulate self-referential thinking during their rest after movie watching. Scan parameters and preprocessing steps were the same as in Study 2. We also administered the Korean version of the RRS. **(a)** After each resting-state run, we asked participants a few questions about their cognitive and affective states during the run. As the plots show, participants (n = 60) had significantly higher levels of self-relevant thought and alertness. Statistical significance was calculated with a paired t-test (Alertness: $t(59) = 6.44$, p = 1.20e-8, one-tailed; Self-relevance: $t(59) = 1.76$, $p = 0.042$, one-tailed). Bounds of boxes indicate 1st and 3rd quartiles, whiskers indicate minima and maxima, and red lines indicate median values. **(b)** Our model showed a significant prediction of depressive rumination only with the post-movie resting-state data, $r = 0.228$ ($p = 0.040$, one-sided permutation test, 95% CI [-0.028, 0.492]). With the pre-movie resting-state data, our model showed non-significant prediction, $r = -0.038$ ($p = 0.613$, one-sided permutation test, 95% CI [-0.298, 0.222]). Each dot indicates a depressive rumination score and a model response of the single participant.

Supplementary Table 1. Demographic information and self-report questionnaires

Variables	Study 1 (*n* = 84)	Study 2 (*n* = 61)[b]	Study 3 (*n* = 48)[b]	Study 4 (*n* = 35)[c]
Demographics				
Age	28.0 ± 4.9[a]	22.9 ± 2.5	22.8 ± 2.4	44.1 ± 12.1
Gender (M/F)	41 / 43	31 / 30	28 / 20	18 / 17
Self-report questionnaires				
RRS Brooding	7.6 ± 2.5	11.7 ± 4.8	12.6 ± 4.3	-
RRS Depressive rumination	16.7 ± 5.7	13.0 ± 4.3	13.8 ± 4.3	-
RRS Reflective pondering	7.4 ± 2.8	11.9 ± 4.2	12.4 ± 3.5	-
BDI Sum	6.6 ± 6.8	-	-	25.2 ± 9.0
CES-D	-	12.6 ± 8.7	13.7 ± 7.2	-
STAI-X2	-	21.1 ± 10.2	24.1 ± 9.5	-
STAI-X1	-	39.1 ± 6.8	39.8 ± 7.8	-

Note. [a] Mean ± Standard deviation. [b] Studies 2 and 3 used Korean translation of the self-report questionnaires. [c] Study 4 used Japanese translation of the self-report questionnaire

Supplementary Table 2. Training and testing results of all models.

Seed	Training (*n* = 84)						Test1 (*n* = 61)						Test2 (n = 48)	
	Brood		Depressive		Reflective		Brood		Depressive		Reflective		Depressive	
	corr	95% CI	corr	95% CI	corr	95% CI	corr	95% CI	corr	95% CI	corr	95% CI	corr	95% CI
**dMPFC**	-0.114	[-0.332 0.103]	**0.342**	**[0.139 0.574]**	0.062	[-0.156 0.280]			**0.240**	**[-0.013 0.502]**			**0.288**	**[0.004 0.589]**
**vMPFC**	**0.288**	**[0.079 0.514]**	-0.180	[-0.400 0.036]	0.015	[-0.203 0.233]	0.131	[-0.125 0.389]						
HF (L)	-0.284	[-0.510 -0.074]	0.071	[-0.147 0.289]	-0.058	[-0.276 0.160]								
HF (R)	0.014	[-0.204 0.232]	-0.118	[-0.336 0.099]	-0.170	[-0.389 0.046]								
LTC (L)	0.052	[-0.166 0.270]	-0.106	[-0.324 0.111]	0.107	[-0.110 0.325]								
**PCC (L)**	**0.392**	**[0.196 0.632]**	0.042	[-0.176 0.260]	-0.046	[-0.264 0.172]	-0.109	[-0.367 0.148]						
PHC (L)	0.195	[-0.020 0.415]	0.096	[-0.121 0.314]	-0.008	[-0.226 0.210]								
Rsp (L)	-0.116	[-0.334 0.101]	0.289	[0.080 0.515]	0.259	[0.047 0.483]								
TPJ (L)	0.023	[-0.195 0.241]	-0.007	[-0.225 0.211]	0.221	[0.007 0.443]								
LTC (R)	0.166	[-0.050 0.385]	-0.140	[-0.359 0.077]	-0.280	[-0.505 -0.070]								
PCC (R)	-0.069	[-0.287 0.149]	0.133	[-0.084 0.352]	0.158	[-0.058 0.377]								
**PHC (R)**	0.007	[-0.211 0.225]	0.123	[-0.094 0.341]	**0.450**	**[0.267 0.703]**					0.131	[-0.125 0.388]		
Rsp (R)	-0.231	[-0.453 -0.018]	-0.281	[-0.506 -0.071]	-0.179	[-0.399 0.037]								
**TPJ (R)**	-0.153	[-0.372 0.064]	-0.102	[-0.320 0.115]	0.564	[0.421 0.857]					0.184	[-0.072 0.443]		
pIPL (L)	0.117	[-0.100 0.335]	-0.135	[-0.354 0.082]	0.032	[-0.186 0.250]								
**TempP (L)**	**0.314**	**[0.107 0.543]**	0.009	[-0.209 0.227]	**0.294**	**[0.085 0.521]**	-0.045	[-0.302 0.212]			-0.022	[-0.280 0.235]		
aMPFC (L)	0.101	[-0.116 0.319]	0.235	[0.022 0.457]	-0.165	[-0.384 0.051]								
pIPL (R)	0.126	[-0.091 0.344]	-0.039	[-0.257 0.179]	0.237	[0.024 0.459]								
TempP (R)	-0.067	[-0.285 0.151]	-0.121	[-0.339 0.096]	-0.114	[-0.332 0.103]								
aMPFC (R)	0.082	[-0.136 0.300]	0.232	[0.018 0.454]	0.250	[0.038 0.473]								

Note. This table provides 95% confidence intervals of correlations reported in **Table 1.**

Supplementary Table 3. Training and testing results using static connectivity.

Seed	Training (*n* = 84)									Test1 (*n* = 61)					
	Brood			Depressive			Reflective			Brood			Depressive		
	corr	permP	95% CI	corr	permP	95% CI	corr	permP	95% CI	corr	permP	95% CI	corr	permP	95% CI
dMPFC	0.087	0.214	[-0.131 0.305]	0.131	0.116	[-0.086 0.350]	0.221	0.022	[0.007 0.442]						
vMPFC	0.018	0.440	[-0.200 0.236]	0.129	0.122	[-0.088 0.347]	-0.344	0.999	[-0.576 -0.141]						
HF (L)	0.037	0.371	[-0.181 0.255]	0.101	0.188	[-0.116 0.319]	0.024	0.414	[-0.194 0.242]						
HF (R)	-0.032	0.615	[-0.250 0.186]	-0.063	0.723	[-0.281 0.155]	-0.073	0.748	[-0.291 0.145]						
LTC (L)	0.158	0.074	[-0.058 0.377]	0.040	0.360	[-0.178 0.258]	-0.248	0.989	[-0.471 -0.036]						
PCC (L)	0.107	0.167	[-0.110 0.325]	-0.185	0.954	[-0.405 0.031]	-0.193	0.959	[-0.413 0.022]						
PHC (L)	0.079	0.234	[-0.139 0.297]	-0.130	0.883	[-0.349 0.087]	-0.006	0.518	[-0.224 0.212]						
Rsp (L)	0.142	0.098	[-0.075 0.361]	-0.065	0.716	[-0.283 0.153]	-0.175	0.942	[-0.395 0.041]						
TPJ (L)	0.052	0.326	[-0.166 0.270]	-0.113	0.847	[-0.331 0.104]	-0.200	0.967	[-0.421 0.015]						
LTC (R)	0.244	0.017	[0.031 0.467]	-0.175	0.944	[-0.395 0.041]	-0.109	0.838	[-0.327 0.108]						
PCC (R)	0.284	0.005	[0.074 0.510]	-0.034	0.623	[-0.252 0.184]	0.045	0.346	[-0.173 0.263]						
PHC (R)	-0.085	0.778	[-0.303 0.133]	-0.187	0.955	[-0.407 0.029]	-0.287	0.996	[-0.513 -0.078]						
Rsp (R)	-0.268	0.992	[-0.492 -0.057]	-0.277	0.995	[-0.502 -0.067]	-0.351	1.000	[-0.584 -0.149]						
TPJ (R)	-0.122	0.862	[-0.340 0.095]	-0.031	0.614	[-0.249 0.187]	-0.082	0.769	[-0.300 0.136]						
**pIPL (L)**	**0.418**	**0.000**	**[0.227 0.663]**	**0.377**	**0.000**	**[0.179 0.614]**	0.262	0.010	[0.050 0.486]	-0.039	0.615	[-0.233 0.281]	0.024	0.432	[-0.296 0.218]
TempP (L)	0.219	0.019	[0.005 0.440]	-0.088	0.786	[-0.306 0.130]	0.234	0.017	[0.021 0.456]						
aMPFC (L)	-0.071	0.737	[-0.289 0.147]	-0.119	0.859	[-0.337 0.098]	0.054	0.309	[-0.164 0.272]						
pIPL (R)	-0.093	0.796	[-0.311 0.125]	-0.116	0.854	[-0.334 0.101]	-0.090	0.788	[-0.308 0.128]						
TempP (R)	0.234	0.014	[0.021 0.456]	0.151	0.088	[-0.066 0.370]	0.281	0.006	[0.071 0.507]						
aMPFC (R)	0.192	0.041	[-0.023 0.412]	0.176	0.053	[-0.040 0.396]	0.191	0.038	[-0.024 0.411]						

Note. Using the 20 regions-of-interest (ROIs) within the default mode network, we trained and tested predictive models using static functional connectivity as input features. We corrected the significance for the multiple tests with the false discovery rate (FDR) $q < .05$ ($p <$ 1e-4). All *p*-values are from one-sided permutation test. (L): Left; (R): Right. dmPFC: Dorsomedial prefrontal cortex, vmPFC: Ventromedial prefrontal cortex, HF: Hippocampal formation, LTC: Lateral temporal cortex, PCC: Posterior cingulate cortex, PHC: Parahippocampal cortex. Rsp: Retrosplenial cortex, TPJ: Temporoparietal junction, pIPL: posterior inferior parietal lobule, TempP: Temporal pole, aMPFC: anterior medial prefrontal cortex.

Supplementary Table 4. Prediction results using Study 2 as a training dataset.

Seed	Training (*n* = 61)									Test1 (*n* = 84)								
	Brood			Depressive			Reflective			Brood			Depressive			Depressive		
	corr	permP	95% CI	corr	permP	95% CI	corr	permP	95% CI	corr	permP	95% CI	corr	permP	95% CI	corr	permP	95% CI
**dMPFC**	0.191	0.072	[-0.024 0.411]	-0.142	0.862	[-0.400 0.114]	**0.396**	**0.001**	**[0.162 0.676]**							0.042	0.351	[-0.176 0.260]
**vMPFC**	**0.398**	**0.001**	**[0.203 0.639]**	-0.173	0.907	[-0.432 0.083]	0.007	0.467	[-0.250 0.264]	0.066	0.273	[-0.152 0.284]						
**HF (L)**	0.091	0.241	[-0.127 0.309]	0.025	0.422	[-0.232 0.282]	**0.429**	**0.001**	**[0.201 0.716]**							-0.216	0.978	[-0.437 -0.002
HF (R)	-0.201	0.941	[-0.422 0.014]	-0.304	0.991	[-0.571 -0.057]	-0.209	0.954	[-0.469 0.045]									
LTC (L)	0.209	0.053	[-0.006 0.430]	0.048	0.349	[-0.209 0.305]	-0.211	0.946	[-0.472 0.043]									
PCC (L)	-0.039	0.625	[-0.257 0.179]	-0.098	0.775	[-0.356 0.159]	-0.139	0.848	[-0.397 0.117]									
**PHC (L)**	-0.198	0.937	[-0.418 0.017]	-0.227	0.964	[-0.488 0.026]	**0.366**	**0.001**	**[0.126 0.641]**							-0.076	0.762	[-0.294 0.142]
Rsp (L)	0.274	0.015	[0.063 0.499]	0.040	0.383	[-0.217 0.297]	-0.155	0.884	[-0.414 0.101]									
TPJ (L)	0.193	0.068	[-0.022 0.413]	0.103	0.216	[-0.154 0.361]	-0.079	0.720	[-0.337 0.178]									
LTC (R)	-0.276	0.984	[-0.501 -0.066]	0.171	0.097	[-0.085 0.430]	-0.100	0.787	[-0.358 0.157]									
PCC (R)	-0.204	0.943	[-0.425 0.011]	0.175	0.089	[-0.081 0.434]	-0.223	0.959	[-0.484 0.031]									
PHC (R)	0.060	0.339	[-0.158 0.278]	0.302	0.009	[0.054 0.569]	0.258	0.022	[0.007 0.521]									
**Rsp (R)**	0.154	0.113	[-0.063 0.373]	0.009	0.470	[-0.248 0.266]	**0.384**	**0.001**	**[0.147 0.662]**							-0.12	0.861	[-0.338 0.097]
TPJ (R)	0.237	0.032	[0.024 0.459]	0.308	0.009	[0.061 0.576]	-0.231	0.966	[-0.493 0.022]									
**pIPL (L)**	-0.059	0.673	[-0.277 0.159]	0.157	0.113	[-0.099 0.416]	**0.349**	**0.004**	**[0.107 0.622]**							-0.17	0.938	[-0.389 0.046]
**TempP (L)**	0.172	0.090	[-0.044 0.392]	**0.323**	**0.004**	**[0.078 0.592]**	**0.442**	**0.000**	**[0.217 0.732]**				-0.002	0.511	[-0.220 0.216]	0.078	0.238	[-0.140 0.296]
**aMPFC (L)**	-0.076	0.713	[-0.294 0.142]	**0.334**	**0.004**	**[0.090 0.605]**	0.226	0.038	[-0.027 0.487]				-0.036	0.617	[-0.254 0.182]			
pIPL (R)	-0.101	0.783	[-0.319 0.116]	-0.002	0.518	[-0.259 0.255]	-0.057	0.661	[-0.314 0.200]									
TempP (R)	0.208	0.054	[-0.007 0.429]	-0.205	0.936	[-0.465 0.049]	0.201	0.059	[-0.054 0.461]									
aMPFC (R)	0.080	0.273	[-0.138 0.298]	0.262	0.021	[0.011 0.526]	-0.006	0.519	[-0.263 0.251]									

Note. Using the same 20 default mode network regions-of-interest as in Table 1, we trained and tested the models using the variance of seed-based dynamic functional connectivity. The difference is that this shows the results of using Study 2 as a training dataset. We corrected for multiple comparisons with the false discovery rate ($p < .009$ for FDR $q < .05$). Here, we used Study 1 dataset for validation. All p-values are from one-sided permutation test. (L): Left; (R): Right. dmPFC: Dorsomedial prefrontal cortex, vmPFC: Ventromedial prefrontal cortex, HF: Hippocampal formation, LTC: Lateral temporal cortex, PCC: Posterior cingulate cortex, PHC: Parahippocampal cortex. Rsp: Retrosplenial cortex, TPJ: Temporoparietal junction, pIPL: posterior inferior parietal lobule, TempP: Temporal pole, amPFC: anterior medial prefrontal cortex.

Supplementary Table 5. Prediction results using Study 3 as a training dataset.

Seed	Training (n = 48)									Test1 (n = 84)						Test2 (n = 61)		
	Brood			Depressive			Reflective			Brood			Reflective			Brood		
	corr	permP	95% CI	corr	permP	95% CI	corr	permP	95% CI	corr	permP	95% CI	corr	permP	95% CI	corr	permP	95% CI
dMPFC	0.009	0.473	[-0.248 0.266]	-0.056	0.656	[-0.313 0.201]	0.158	0.144	[-0.098 0.417]									
vMPFC	0.411	0.002	[0.179 0.694]	0.215	0.073	[-0.039 0.476]	-0.175	0.880	[-0.434 0.081]									
HF (L)	-0.108	0.771	[-0.366 0.149]	-0.004	0.513	[-0.261 0.253]	0.110	0.224	[-0.147 0.368]									
**HF (R)**	**0.527**	**0.000**	**[0.329 0.843]**	0.152	0.155	[-0.104 0.411]	-0.083	0.721	[-0.341 0.174]	**0.187**	**0.041**	**[-0.029 0.407]**				-0.067	0.692	[-0.325 0.190]
LTC (L)	-0.150	0.845	[-0.408 0.106]	-0.195	0.911	[-0.455 0.060]	0.292	0.022	[0.043 0.558]									
PCC (L)	-0.478	1.000	[-0.778 -0.263]	0.101	0.250	[-0.156 0.359]	0.390	0.004	[0.154 0.669]									
**PHC (L)**	0.289	0.023	[0.040 0.555]	0.145	0.169	[-0.111 0.403]	**0.436**	**0.002**	**[0.210 0.725]**				0.075	0.249	[-0.143 0.293]			
Rsp (L)	-0.306	0.983	[-0.573 -0.059]	-0.194	0.903	[-0.454 0.061]	-0.323	0.989	[-0.592 -0.078]									
TPJ (L)	0.025	0.432	[-0.232 0.282]	0.082	0.297	[-0.175 0.340]	-0.414	0.999	[-0.698 -0.183]									
LTC (R)	-0.143	0.835	[-0.401 0.113]	-0.280	0.971	[-0.545 -0.030]	0.077	0.297	[-0.180 0.335]									
PCC (R)	0.351	0.007	[0.109 0.624]	-0.285	0.975	[-0.550 -0.036]	-0.221	0.934	[-0.482 0.033]									
PHC (R)	0.064	0.332	[-0.193 0.321]	-0.290	0.980	[-0.556 -0.041]	-0.515	1.000	[-0.827 -0.312]									
Rsp (R)	-0.043	0.610	[-0.300 0.214]	0.339	0.008	[0.096 0.610]	0.088	0.263	[-0.169 0.346]									
TPJ (R)	0.026	0.422	[-0.231 0.283]	-0.111	0.777	[-0.369 0.146]	-0.227	0.937	[-0.488 0.026]									
pIPL (L)	0.344	0.009	[0.101 0.616]	-0.136	0.816	[-0.394 0.121]	0.264	0.037	[0.013 0.528]									
TempP (L)	-0.042	0.620	[-0.299 0.215]	0.004	0.490	[-0.253 0.261]	-0.421	0.999	[-0.706 -0.192]									
aMPFC (L)	0.160	0.139	[-0.096 0.419]	-0.207	0.914	[-0.467 0.047]	0.094	0.262	[-0.163 0.352]									
pIPL (R)	-0.348	0.992	[-0.621 -0.106]	0.162	0.137	[-0.094 0.421]	0.217	0.065	[-0.037 0.478]									
TempP (R)	-0.285	0.973	[-0.550 -0.036]	-0.195	0.909	[-0.455 0.060]	-0.132	0.813	[-0.390 0.125]									
aMPFC (R)	-0.091	0.734	[-0.349 0.166]	-0.072	0.680	[-0.329 0.185]	-0.214	0.934	[-0.475 0.040]									

Note. Same as **Supplementary Table 4**, except that this table shows the results of using Study 3 as a training dataset.

Supplementary Table 6. Training and testing results using the number of features same as the original model, n_{feature} = 84

Seed	Training (*n* = 109; Studies 2 and 3)									Test (*n* = 84; Study 1)					
	Brood			Depressive			Reflective			Brood			Depressive		
	corr	permP	95% CI	corr	permP	95% CI	corr	permP	95% CI	corr	permP	95% CI	corr	permP	95% CI
**dMPFC**	-0.016	0.561	[-0.273 0.241]	**0.275**	**0.002**	**[0.025 0.540]**	-0.069	0.767	[-0.326 0.188]				**0.190**	**0.042**	**[-0.025 0.410]**
**vMPFC**	**0.255**	**0.003**	**[0.003 0.518]**	-0.154	0.948	[-0.413 0.102]	0.186	0.029	[-0.069 0.446]	-0.150	0.916	[-0.379 0.067]			
HF (L)	-0.045	0.687	[-0.302 0.212]	-0.117	0.885	[-0.375 0.140]	0.001	0.487	[-0.256 0.258]						
HF (R)	-0.169	0.961	[-0.428 0.087]	0.024	0.411	[-0.233 0.281]	-0.009	0.539	[-0.266 0.248]						
**LTC (L)**	0.018	0.420	[-0.239 0.275]	**0.388**	**0.000**	**[0.152 0.667]**	-0.117	0.891	[-0.375 0.140]				-0.171	0.940	[-0.391 0.045]
PCC (L)	0.034	0.366	[-0.223 0.291]	0.032	0.367	[-0.225 0.289]	0.206	0.017	[-0.048 0.466]						
PHC (L)	-0.001	0.509	[-0.258 0.256]	0.172	0.035	[-0.084 0.431]	0.006	0.470	[-0.251 0.263]						
**Rsp (L)**	**0.264**	**0.002**	**[0.013 0.528]**	-0.040	0.654	[-0.297 0.217]	0.106	0.138	[-0.151 0.364]	**0.203**	**0.034**	**[-0.120 0.424]**			
TPJ (L)	-0.014	0.557	[-0.271 0.243]	-0.062	0.741	[-0.319 0.195]	-0.112	0.875	[-0.370 0.145]						
LTC (R)	-0.014	0.551	[-0.271 0.243]	-0.068	0.756	[-0.325 0.189]	-0.150	0.942	[-0.408 0.106]						
PCC (R)	0.089	0.177	[-0.168 0.347]	-0.020	0.570	[-0.277 0.237]	-0.025	0.604	[-0.282 0.232]						
PHC (R)	0.104	0.142	[-0.153 0.362]	-0.046	0.683	[-0.303 0.211]	-0.192	0.978	[-0.452 0.063]						
Rsp (R)	-0.217	0.988	[-0.478 0.037]	-0.014	0.549	[-0.271 0.243]	0.063	0.258	[-0.194 0.320]						
TPJ (R)	-0.001	0.505	[-0.258 0.256]	0.125	0.101	[-0.132 0.383]	0.015	0.437	[-0.242 0.272]						
pIPL (L)	0.101	0.152	[-0.156 0.359]	-0.091	0.820	[-0.349 0.166]	-0.042	0.669	[-0.299 0.215]						
TempP (L)	-0.086	0.808	[-0.344 0.171]	0.145	0.072	[-0.111 0.403]	0.091	0.178	[-0.166 0.349]						
aMPFC (L)	0.094	0.161	[-0.163 0.352]	0.089	0.181	[-0.168 0.347]	0.001	0.500	[-0.256 0.258]						
pIPL (R)	0.054	0.291	[-0.203 0.311]	-0.006	0.522	[-0.263 0.251]	-0.038	0.650	[-0.295 0.219]						
**TempP (R)**	0.068	0.241	[-0.189 0.325]	**0.299**	**0.000**	**[0.051 0.566]**	-0.014	0.559	[-0.271 0.243]				0.143	0.095	[-0.074 0.362]
aMPFC (R)	0.131	0.082	[-0.126 0.389]	0.039	0.341	[-0.218 0.296]	0.049	0.301	[-0.208 0.306]						

Note. This table shows the results using the combined dataset of Studies 2 and 3 (*n* = 109) as the training dataset and the Study 1 dataset (*n* = 84) as the testing dataset. We used the same 20 default mode network regions-of-interest as in Table 1 and trained and tested the models using the variance of seed-based dynamic functional connectivity. We corrected for multiple comparisons with the false discovery rate ($p < .003$ for FDR $q < .05$). All *p*-values are from the one-sided permutation test.

Supplementary Table 7. Training and testing results using the maximum number of features, $n_{feature}$ = 109

Seed	Training (*n* = 109; Studies 2 and 3)									Test (*n* = 84; Study 1)								
	Brood			Depressive			Reflective			Brood			Depressive			Reflective		
	corr	permP	95% CI	corr	permP	95% CI	corr	permP	95% CI	corr	permP	95% CI	corr	permP	95% CI	corr	permP	95% CI
**dMPFC**	0.066	0.255	[-0.191 0.323]	**0.244**	**0.005**	**[-0.008 0.506]**	-0.089	0.821	[-0.347 0.168]				**0.207**	**0.029**	**[-0.008 0.428]**			
vMPFC	0.189	0.026	[-0.066 0.449]	-0.155	0.949	[-0.414 0.101]	0.135	0.087	[-0.122 0.393]									
HF (L)	-0.084	0.814	[-0.342 0.173]	-0.118	0.888	[-0.376 0.139]	0.016	0.431	[-0.241 0.273]									
HF (R)	-0.136	0.923	[-0.394 0.121]	-0.012	0.551	[-0.269 0.245]	0.000	0.506	[-0.257 0.257]									
**LTC (L)**	0.068	0.237	[-0.189 0.325]	**0.391**	**0.000**	**[0.156 0.670]**	-0.029	0.625	[-0.286 0.228]				-0.175	0.944	[-0.395 0.041]			
**PCC (L)**	0.077	0.211	[-0.180 0.335]	-0.016	0.563	[-0.273 0.241]	**0.255**	**0.004**	**[0.003 0.518]**							0.161	0.077	[-0.055 0.380]
PHC (L)	0.011	0.460	[-0.246 0.268]	0.176	0.030	[-0.080 0.435]	0.069	0.235	[-0.188 0.326]									
**Rsp (L)**	**0.273**	**0.001**	**[0.023 0.537]**	-0.030	0.625	[-0.287 0.227]	0.107	0.130	[-0.150 0.365]	0.159	0.075	[-0.057 0.378]						
TPJ (L)	-0.021	0.587	[-0.278 0.236]	-0.040	0.661	[-0.297 0.217]	-0.096	0.834	[-0.354 0.161]									
LTC (R)	-0.044	0.668	[-0.301 0.213]	-0.092	0.829	[-0.350 0.165]	-0.109	0.871	[-0.367 0.148]									
PCC (R)	0.102	0.143	[-0.155 0.360]	-0.080	0.792	[-0.338 0.177]	-0.037	0.650	[-0.294 0.220]									
PHC (R)	0.073	0.226	[-0.184 0.330]	-0.124	0.904	[-0.382 0.133]	-0.166	0.962	[-0.425 0.090]									
Rsp (R)	-0.231	0.992	[-0.493 0.022]	-0.052	0.700	[-0.309 0.205]	0.046	0.317	[-0.211 0.303]									
TPJ (R)	0.008	0.464	[-0.249 0.265]	0.182	0.029	[-0.073 0.441]	-0.093	0.831	[-0.351 0.164]									
pIPL (L)	0.065	0.256	[-0.192 0.322]	-0.020	0.577	[-0.277 0.237]	-0.105	0.864	[-0.363 0.152]									
TempP (L)	-0.106	0.856	[-0.364 0.151]	0.119	0.113	[-0.138 0.377]	0.022	0.416	[-0.235 0.279]									
aMPFC (L)	0.098	0.152	[-0.159 0.356]	0.040	0.336	[-0.217 0.297]	-0.019	0.579	[-0.276 0.238]									
pIPL (R)	0.076	0.212	[-0.181 0.334]	-0.049	0.694	[-0.306 0.208]	-0.023	0.595	[-0.280 0.234]									
**TempP (R)**	0.121	0.105	[-0.136 0.379]	**0.327**	**0.000**	**[0.082 0.597]**	-0.009	0.534	[-0.266 0.248]				0.066	0.271	[-0.152 0.284]			
aMPFC (R)	0.160	0.046	[-0.096 0.419]	0.087	0.183	[-0.170 0.345]	0.083	0.197	[-0.174 0.341]									

Note. Same as **Supplementary Table 6**, except this shows the results of using the maximum possible number of features for prediction.

Supplementary References

1 Yamashita, A. et al. Harmonization of resting-state functional MRI data across multiple imaging sites via the separation of site differences into sampling bias and measurement bias. Plos Biology 17, doi:10.1371/journal.pbio.3000042 (2019).

2 Finn, E. S. Is it time to put rest to rest? Trends in Cognitive Sciences 25, 1021-1032, doi:10.1016/j.tics.2021.09.005 (2021).
