# Supplementary material for: A dorsomedial prefrontal cortex-based dynamic functional connectivity model of rumination
Source: Nat Commun. 2023 Jun 15;14:3540. doi: 10.1038/s41467-023-39142-9 (PMC10272121; doi:10.1038/s41467-023-39142-9)
Supplement: Supplementary file 2 — Reporting Summary [file 41467_2023_39142_MOESM2_ESM.pdf]

nature portfolio

Corresponding author(s): Choong-Wan Woo, Tor.D.Wager

Last updated by author(s): May 11, 2023

Reporting Summary

Nature Portfolio wishes to improve the reproducibility of the work that we publish. This form provides structure for consistency and transparency in reporting. For further information on Nature Portfolio policies, see our Editorial Policies and the Editorial Policy Checklist.

Statistics

For all statistical analyses, confirm that the following items are present in the figure legend, table legend, main text, or Methods section.

n/a	Confirmed	
☐	☒	The exact sample size (*n*) for each experimental group/condition, given as a discrete number and unit of measurement
☐	☒	A statement on whether measurements were taken from distinct samples or whether the same sample was measured repeatedly
☐	☒	The statistical test(s) used AND whether they are one- or two-sided<br>*Only common tests should be described solely by name; describe more complex techniques in the Methods section.*
☐	☒	A description of all covariates tested
☐	☒	A description of any assumptions or corrections, such as tests of normality and adjustment for multiple comparisons
☐	☒	A full description of the statistical parameters including central tendency (e.g. means) or other basic estimates (e.g. regression coefficient) AND variation (e.g. standard deviation) or associated estimates of uncertainty (e.g. confidence intervals)
☐	☒	For null hypothesis testing, the test statistic (e.g. *F*, *t*, *r*) with confidence intervals, effect sizes, degrees of freedom and *P* value noted<br>*Give P values as exact values whenever suitable.*
☒	☐	For Bayesian analysis, information on the choice of priors and Markov chain Monte Carlo settings
☒	☐	For hierarchical and complex designs, identification of the appropriate level for tests and full reporting of outcomes
☐	☒	Estimates of effect sizes (e.g. Cohen's *d*, Pearson's *r*), indicating how they were calculated

Our web collection on statistics for biologists contains articles on many of the points above.

Software and code

Policy information about availability of computer code

Data collection	Matlab (v 2017a, Mathworks) and Psychtoolbox (version 3.0, http://psychtoolbox.org) were used to display the fixation cross.
Data analysis	We analyzed the data using Matlab (v 2017b and v 2020b, Mathworks), SPM v8 & v12, and in-house neuroimaging softwares (CanlabCore [https://github.com/canlab/CanlabCore]) and CocoanCore [https://github.com/cocoanlab/CocoanCore]). For dynamic connectivity analysis, the Dynamic Conditional Correlation toolbox was used (https://github.com/canlab/Lindquist_Dynamic_Correlation). For the codes to generate main figures are available at "https://github.com/cocoanlab/rumination" and "https://doi.org/10.5281/zenodo.7923949".

For manuscripts utilizing custom algorithms or software that are central to the research but not yet described in published literature, software must be made available to editors and reviewers. We strongly encourage code deposition in a community repository (e.g. GitHub). See the Nature Portfolio guidelines for submitting code & software for further information.

Data

Policy information about availability of data

All manuscripts must include a data availability statement. This statement should provide the following information, where applicable:

- Accession codes, unique identifiers, or web links for publicly available datasets
- A description of any restrictions on data availability
- For clinical datasets or third party data, please ensure that the statement adheres to our policy

Data of studies 1-3 and supplementary dataset to generate main results and supplementary information of the study are provided at the following link (https://github.com/cocoanlab/rumination; https://doi.org/10.5281/zenodo.7923949). The data for Study 4 are available at https://bicr-resource.atr.jp/srpbs1600/. Raw

data for studies 1-3 will be available upon request if there are no conflicts of interest between co-authors.

Research involving human participants, their data, or biological material

Policy information about studies with human participants or human data. See also policy information about sex, gender (identity/presentation), and sexual orientation and race, ethnicity and racism.

Reporting on sex and gender	Our purpose of the study was to develop a rumination predictive marker that is generalizable across all populations, including sexes and/or genders. So we recruited an even number of male and female participants as possible based on their self-report. (Female/Male, Study1: 43/41, Study2: 30/31, Study3: 20/28, Study4: 17/18). All participants provided written informed consent for their data sharing, and their reported gender and age are listed in the Source Data file.
Reporting on race, ethnicity, or other socially relevant groupings	- Study 1: 34 Non-Hispanic White Americans, 30 White Americans, and 20 Non-Hispanic African Americans.<br>- Study 2-4, Supplementary data: All Asian participants. Participants from Study 2-3 and Supplementary dataset were Koreans (n = 169) and Participants from Study 4 were Japanese (n = 35).
Population characteristics	- Study 1: healthy and right-handed participants (USA), n = 84 (age = 28.0 ± 4.9 [mean ± SD], 41 males)<br>- Study 2: healthy and right-handed participants (South Korea). n = 61 (age = 22.9 ± 2.5 [mean ± SD], 31 males).<br>- Study 3: healthy and right-handed participants (South Korea). n = 48 (age = 22.8 ± 2.4 [mean ± SD], 28 males).<br>- Study 4: right-handed participants diagnosed with major depression disorder (Japan). n = 35 (age = 44.1 ± 12.1 [mean ± SD], 18 males)<br>- Supplementary data: healthy and right-handed participants (South Korea). n = 60 (age = 23.4 ± 1.9 [mean ± SD], 30 males).
Recruitment	- Study 1: Healthy adults were recruited across the greater Denver area and from existing subject databases assembled by the University of Colorado Boulder Institute for Behavioral Genetics.<br>- Study 2-3: Healthy adults were recruited from Suwon, South Korea.<br>- Study 4: Participants were recruited by University of Hiroshima. (Tanaka et al., 2021, Scientific data)<br>- Supplementary data: Healthy adults were recruited from Suwon, South Korea.<br><br>For study 2-3, and supplementary data, we randomly recruited participants who contacted us via flyers posted across Suwon area. We only excluded participants who were not eligible for the fMRI experiment, who had psychiatric, neurological, or systemic disorders and MRI contraindications.
Ethics oversight	The institutional review board of of the University of Colorado Boulder (Study 1), Sungkyunkwan University (Study 2-3, Supplementary data), and the University of Hiroshima (Study 4) approved the study. All participants in Study 4 provided written informed consent for the study participation and the data sharing after anonymization (Tanaka et al., 2021).

Note that full information on the approval of the study protocol must also be provided in the manuscript.

Field-specific reporting

Please select the one below that is the best fit for your research. If you are not sure, read the appropriate sections before making your selection.

[x] Life sciences [ ] Behavioural & social sciences [ ] Ecological, evolutionary & environmental sciences

For a reference copy of the document with all sections, see nature.com/documents/nr-reporting-summary-flat.pdf

Life sciences study design

All studies must disclose on these points even when the disclosure is negative.

Sample size	The current study included five datasets. Studies 1-3 data, and supplementary data were from a resting-state scan of three independent fMRI studies (Study 1: n = 84, Study 2: n = 61, Study 3: n = 48, Supplementary data: n = 60) from two sites (Study 1: University of Colorado Boulder, USA, Studies 2-3 and Supplementary data: Sungkyunkwan University, South Korea). Study 4 data (n = 35) were from a publicly available dataset (Tanaka et al., 2021), which provides resting-state fMRI data of individuals with multiple psychiatric disorders from multiple sites. Among the sites, we used the data from the Center of Innovation at Hiroshima University, since it had the largest number of individuals with major depression disorder. No statistical method was used to predetermine the sample size, but our result shows generalizable performance in moderate sample size, compared to previous literature that provided generalizable predictive marker (Lee et al., 2021; Spisak et al., 2020).
Data exclusions	In Study 1 (training dataset), we excluded 26 participants whose behavioral data (i.e., RRS score) or resting-state fMRI data were missing. In Studies 2-3 (validation and independent test datasets), no participants were excluded. In Study 4 (clinical dataset), we excluded participants who were 1) left handed, and 2) showed temporal mean frame-wise displacement over 0.25 1) to match the hand dominance with participants in Studies 1-3, and 2) to preserve the data quality from excessive movement. In the supplementary dataset, we excluded 4 participants who rated their alertness scores as zero in either of the two resting-state runs, through which we assumed participants were asleep.
Replication	We used the Study 2 (n = 61) dataset for validation and the Study 3 (n = 48) and Study 4 (n = 35) datasets for independent testing, which showed generalizable performance in all of them. We also applied the model in two resting-state runs in the supplementary dataset (n = 60), which showed generalizable performance only in the post-movie watching run.
Randomization	No randomization procedure was used since all participants were scanned under resting-state. Since our purpose was to predict the RRS

	scores themselves, no covariates were controlled.
Blinding	No blinding procedure was used since there were no group variables with respect to behavioral scores.

Reporting for specific materials, systems and methods

We require information from authors about some types of materials, experimental systems and methods used in many studies. Here, indicate whether each material, system or method listed is relevant to your study. If you are not sure if a list item applies to your research, read the appropriate section before selecting a response.

Materials & experimental systems

n/a	Involved in the study
☒	☐ Antibodies
☒	☐ Eukaryotic cell lines
☒	☐ Palaeontology and archaeology
☐	☒ Animals and other organisms
☒	☐ Clinical data
☒	☐ Dual use research of concern
☒	☐ Plants

Methods

n/a	Involved in the study
☒	☐ ChIP-seq
☒	☐ Flow cytometry
☐	☒ MRI-based neuroimaging

Animals and other research organisms

Policy information about studies involving animals; ARRIVE guidelines recommended for reporting animal research, and Sex and Gender in Research

Laboratory animals	n/a
Wild animals	n/a
Reporting on sex	n/a
Field-collected samples	n/a
Ethics oversight	n/a

Note that full information on the approval of the study protocol must also be provided in the manuscript.

Magnetic resonance imaging

Experimental design

Design type	Resting-state
Design specifications	We used a single run from each dataset (Study 1 - 4) lasting 7 minutes, 6 minutes, 6 minutes, and 10 minutes, respectively. Participants were asked to stare at the fixation point during the scan. In Study 3, participants were intermittently asked to report their momentary thoughts in a word or in a phrase, which was not used in the current study. We also used two 14 minutes of resting-state runs in the supplementary dataset.
Behavioral performance measures	We administered a set of self-report questionnaires outside of the scanner. For the supplementary dataset, we administered in-scanner reports, through which we asked participants to rate their alertness and self-relevance of thoughts they had during the run.

Acquisition

Imaging type(s)	Functional, Structural
Field strength	3T
Sequence & imaging parameters	- Study 1 : Functional MRI (3×3×3 mm voxels, TR: 460 ms, TE: 29 ms, slices: 56, multiband factor=8, flip angle: 44°, FoV read: 248 mm, echo spacing: 0.51 ms, bandwidth: 2772 Hz/Px, time: 10:15). High-resolution T1-weighted structural images were acquired.<br>- Study 2-3, Supplementary data: Functional MRI (2.7×2.7×2.7 mm voxels, TR: 460 ms, TE: 27.2 ms, slices: 56, multiband factor=8, FoV read: 220 mm, 82 x 82 matrix). High-resolution T1-weighted structural images were acquired.<br>- Study 4: Functional MRI (3.3×3.3×3.2 mm voxels, TR: 2500 ms, TE: 30 ms, ascending slices: 40 , FoV read: 212 mm, 64 x 64 matrix). High-resolution T1-weighted structural images were acquired.
Area of acquisition	Whole brain scan.

Diffusion MRI ☐ Used ☒ Not used

Preprocessing

Preprocessing software	- Study 1: We conducted preprocessing steps with the in-house tool mainly based on SPM8 (https://github.com/canlab/preprocess). Functional images were realigned to the first image after discarding initial images and interpolated to 2x2x2 mm3. Smoothing was done with 8-mm FWHM gaussian kernel.<br>- Studies 2-4, Supplementary data: We conducted preprocessing steps with the in-house tool mainly based on SPM12 and FSL (https://github.com/cocoanlab/humanfmri_preproc_bids). Functional images were realigned to the first image after discarding initial images and interpolated to 2x2x2 mm3. Smoothing was done with 5-mm FWHM gaussian kernel.
Normalization	Functional EPI data were normalized to MNI template using non-linear transformation.
Normalization template	MNI 152
Noise and artifact removal	Twenty-four head motion parameters (6 movement parameters including x, y, z, roll, pitch, and yaw, their mean-centered squares, their derivatives, and squared derivatives) were included as nuisance covariates. Five principal component scores from each cerebrospinal fluid signals and white matter signals, and linear drift were also included as nuisance covariates. For Study 3, onsets of thought report (see Experimental Design section above) convolved with hemodynamic response function was additionally included as nuisance covariates. Nuisance covariates were regressed out from preprocessed fMRI data.
Volume censoring	Outlier volumes identified based on mean signal intensity, mahalanobis distances, and root mean square of successive differences were regressed out.

Statistical modeling & inference

Model type and settings	We used LASSO regression for the prediction of the RRS score.
Effect(s) tested	We calculated Pearson's correlation between model predicted and actual scores to evaluate models' prediction performance.

Specify type of analysis: ☐ Whole brain ☐ ROI-based ☒ Both

Anatomical location(s)	We used 20 seed regions in the default mode network specified in Andrews-Hanna et al. (2010) for seed-based connectivity analysis. We also used the whole-brain Brainnetome atlas (Fan et al., 2016).
Statistic type for inference (See Eklund et al. 2016)	For significance testing of model performance, which was based on Pearson's correlation, we conducted permutation tests by shuffling participant labels for the actual and predicted RRS scores with 10,000 iterations.
Correction	To compare multiple model performance, we used false discovery rate (FDR) q < .05 to correct for multiple comparisons.

Models & analysis

n/a	Involved in the study
☐	☒ Functional and/or effective connectivity
☒	☐ Graph analysis
☐	☒ Multivariate modeling or predictive analysis

Functional and/or effective connectivity	We used the Dynamic Condition Correlation (DCC) to estimate the dynamics functional connectivity between brain regions.
Multivariate modeling and predictive analysis	With the averaged time-series in each brain parcel defined by the Brainnetome atlas, we calculated DCC between each default mode network seed region and the brain parcels. Then, we used variance of DCC values as input features for predictive modeling. We used least absolute shrinkage selector operator (LASSO) regression to predict individual RRS scores. After the model validation and independent testing, we conducted the virtual lesion analysis to extract important features of the model. With the refined model with the virtual lesion analysis results, we tested the predictive model on the clinical dataset. All predictive performance was measured based on Pearson's correlation coefficient.
